# Beyond Stereotypes: Analyzing Gender and Cultural Differences in Nonverbal Rapport

**DOI:** 10.3389/fpsyg.2020.599703

**Published:** 2020-12-11

**Authors:** Gary Bente, Eric Novotny, Daniel Roth, Ahmad Al-Issa

**Affiliations:** ^1^Department of Communication, Michigan State University, East Lansing, MI, United States; ^2^Grady College of Journalism and Mass Communication, University of Georgia, Athens, GA, United States; ^3^Department of Computer Aided Medical Procedures and Augmented Reality, Technical University of Munich, Munich, Germany; ^4^Department of English, American University of Sharjah, Sharjah, United Arab Emirates

**Keywords:** rapport, nonverbal behavior, motion capture, character animation, gender, culture

## Abstract

The current paper addresses two methodological problems pertinent to the analysis of observer studies in nonverbal rapport and beyond. These problems concern: (1) the production of standardized stimulus materials that allow for unbiased observer ratings and (2) the objective measurement of nonverbal behaviors to identify the dyadic patterns underlying the observer impressions. We suggest motion capture and character animation as possible solutions to these problems and exemplarily apply the novel methodology to the study of gender and cultural differences in nonverbal rapport. We compared a Western, individualistic culture with an egalitarian gender-role conception (Germany) and a collectivistic culture with a more traditional gender role conceptions (Middle East, Gulf States). Motion capture data were collected for five male and five female dyadic interactions in each culture. Character animations based on the motion capture data served as stimuli in the observation study. Female and male observers from both cultures rated the perceived rapport continuously while watching the 1 min sequences and guessed gender and cultural background of the dyads after each clip. Results show that masking of gender and culture in the stimuli was successful, as hit rates for both aspects remained at chance level. Further the results revealed high levels of agreement in the rapport ratings across gender and culture, pointing to universal judgment policies. A 2 × 2 × 2 × 2 ANOVA for gender and culture of stimuli and observers showed that female dyads were rated significantly higher on rapport across the board and that the contrast between female and male dyads was more pronounced in the Arab sample as compared to the German sample. nonverbal parameters extracted from the motion capture protocols were submitted to a series of algorithms to identify dyadic activity levels and coordination patterns relevant to the perception of rapport. The results are critically discussed with regard to the role of nonverbal coordination as a constituent of rapport.

## Introduction

Communication is a complex and highly demanding task. It can unfold in a harmonious and effortless way, yet sometimes also fail catastrophically. A most critical determinant of communication success is whether the partners “click” on a nonverbal level, or in other words whether they can establish rapport (Granitz et al., 2009). In general terms, rapport is characterized as being connected, tuned-in, or in-sync (Bernieri, 1988). Rapport has been shown to positively influence communication outcomes in a variety of situations, including classroom interactions (Bernieri, 1988; Murphy and Valdéz, 2005; Nguyen, 2007), conflict resolution (Drolet and Morris, 2000), child care (Burns, 1984), therapeutic interventions (Hall et al., 1995; Cooper and Tauber, 2005) business interactions (Gremler and Gwinner, 1998, 2000; Macintosh, 2009), among others. Importantly, rapport is described as an emergent social phenomenon only observable in interactions, defining the dyad as the smallest unit of analysis (Tickle-Degnen and Rosenthal, 1990; Bernieri and Gillis, 1995b). Rapport has been shown to rely on a dyad’s nonverbal expressiveness (Tickle-Degnen, 2006), comprising signals of mutual attentiveness, the reciprocal exchange of positivity cues, and most importantly, the coordination of nonverbal behaviors (Tickle-Degnen and Rosenthal, 1987, 1990; Bernieri, 1988; Bernieri et al., 1996; Grahe and Bernieri, 1999). These coordination patterns include both temporal entrainment (synchrony; Lakin and Chartrand, 2003; Lakens and Stel, 2011; Ramseyer and Tschacher, 2011; Fujiwara and Daibo, 2016) and similarities in form (motor and postural mimicry; Bernieri et al., 1994; Lakin and Chartrand, 2003; Miles et al., 2009). In this sense, Grahe and Bernieri (1999) summarize: “…rapport is primarily a physically manifested construct; it is a construct that is visible at the surface and readily apparent” (p. 265).

In fact, observers seem to be able to assess a dyad’s rapport fromnonverbal interactions very swiftly and with considerable consensus(Gillis et al., 1995; Bernieri et al., 1996). However, Bernieri and Gillis (1995a) report that this consensus could not be established between observers and the interactors, which the authors discussed as a validity problem. It is questionable though, whether self-reports were an appropriate criterion for the validity of observer ratings. Evaluations of rapport provided by the interactors are based on the individual feelings. They may depend on many other factors than nonverbal behaviors and movement coordination, such as for instance others’ group membership, perceived attractiveness, similarity and liking. First-person impressions might even be controversial across the interactors and thus difficult to use as a unified criterion. Third-person judgments of rapport, in contrast, are based on observations of the dyad as a whole and could provide a more neutral picture of the emergent dyadic phenomena. Yet, observation data can evidently be flawed by judgment biases and inaccuracies that may affect intuitive judgments (ratings) of perceived rapport as well as descriptive accounts (behavior coding).

The current study addresses two major methodological problems pertinent to observer studies in nonverbal rapport: (1) the production of appropriate stimulus materials that allow one to assess observers’ perceptions of rapport independently from stereotypes and (2) the provision of objective measures of the nonverbal patterns underlying these perceptions independently from observers’ implicit theories. We suggest motion capture technology and character animation as solutions to these problems. To demonstrate the potential of the novel methodology we present a cross-gender, cross culture study to demonstrate how the tools can be effectively used to study individual and group differences in nonverbal rapport and beyond. To achieve a maximal contrast regarding expected cultural differences in the first study of this kind, we compared a Western, individualistic culture with an egalitarian gender-role conception (Germany) and a collectivistic culture with more traditional gender role conceptions (Middle East, Gulf States). We ask (1) whether nonverbal rapport is consistently perceived by observers from different cultures solely based on the perception of dyadic movement patterns and (2) whether observer judgments reveal differences in the levels of rapport that female and male dyads as well as German and Arab dyads are able to achieve. In an exploratory analysis we finally demonstrate how to identify nonverbal interaction patterns in the motion capture protocols that account for perceived differences in rapport.

## Background

We chose gender and cultural differences for this study for two reasons: first, because both factors are under-investigated with regard to nonverbal rapport; second, because both variables are particularly relevant to stereotype activation and judgment bias (Cuddy et al., 2015; Ellemers, 2018) and thus are ideal candidates to demonstrate the advantages of the novel methodology. Only two studies came to our attention that addressed gender differences in nonverbal rapport. Puccinelli et al. (2003) reported that “female observers perceived dyad members to exhibit more rapport-facilitating behavior” (p. 211) than male observers. As the stimulus material consisted in majority of female dyads, an interaction effect between the gender of interactors and observers is likely. The authors concede that the visible gender of the interactors might have selectively primed female observers’ self-stereotype (cf. Cross and Madson, 1997) and in sum led to higher rapport scores for the predominantly female stimuli. Looking at rapport, Bernieri and Gillis (1995a) reported that judgments of rapport correlated with observed “female gestures” (i.e., gestures predominantly shown within female dyads). The specific features of these gestures remain elusive as the relevant behaviors were categorized by human coders, not revealing any details. Further, one might face a potential circularity here between perceptions of rapport and the spotting of particular nonverbal cues (cf. Cappella, 1990; Bente, 2019). The latter study also included observers with different cultural background (i.e., Greek and US American observers watching American dyads). While interobserver correlations across cultures were strong, the study was inconclusive with regard to the behaviors that drove their impressions. The authors hypothesized that both groups unanimously gave “…insufficient weight to valid behavioral predictors of rapport (such as mutual attention, reciprocal positivity and coordination, cf. Tickle-Degnen and Rosenthal, 1990) while relying on the apparently compelling but invalid cues, smiling and expressivity” (p. 115, inserts by the authors in brackets). This interpretation remains speculative as the stimulus materials showed numerous confounds between physical appearance cues of the interactors as well as different nonverbal channels such as facial expressions, gestures, body movements and postures.

Overall, the few existing studies in this domain point to recurrent methodological problems that result from: (1) the use of video stimuli to assess observer impressions (stimulus problem) and (2) the deployment of human coders to collect behavioral data (observer problem, cf. Bente, 2019).

### The Stimulus Problem

Video stimuli as predominantly used in previous observer studies not only show the nonverbal interaction, but also reveal person characteristics such as gender, race, culture, age or attractiveness that might be relevant to stereotype activation and judgment bias (Dion et al., 1990; Stangor and Crandall, 2013). For instance, gender-role stereotypes could lead to the ascription of higher rapport levels to female as compared to male dyads, just because women are expected to put more emphasis on relational harmony than men (see Cross and Madson, 1997). The same holds true for stereotypes about cultures and assumptions regarding their valuing of social harmony and relatedness (Triandis, 1995; Cuddy et al., 2009). Different techniques have been proposed to solve this problem (cf. Bernieri et al., 1994), including the use of point light displays (Johansson, 1973, 1976) or video quantization techniques (Berry et al., 1991, 1992). However, both methods display specific limitations. Quantization techniques used to degrade video images to rougher mosaic patterns in order to obscure physical appearance are not sufficient to completely eliminate clues to gender and culture (see stimulus examples in Bernieri et al., 1994). Point light displays on the other hand fail to capture postural information (see Cutting and Proffitt, 1981; Runeson and Frykholm, 1981). We here suggest the use of computer animations of standardized, neutral characters (avatars). Based on full body motion capture data collected in dyadic interactions such animations allow one to obscure gender, culture and other obvious individual characteristics of the interactors while portraying movements and postures with high fidelity (cf. Bente, 2019).

### The Observer Problem

Implicit theories about relevant rapport indictors cannot only mislead observers’ evaluative impressions (cf. Bernieri and Gillis, 2001), but inversely, observers’ impressions of relational quality can also bias their description of the behavior. Cappella (1990) holds that for instance judgments of coordination “…, whether by participants or observers, could be confounded with judgments of positivity if judges’ implicit theories of social interaction are that positive interactions are ones in which the people are in sync. If this is the case, then the judges would be assessing positivity and not synchrony, and the correlation to rapport would be an artifact” (p. 303). To avoid such circularities, descriptive movement data are needed that are independent from observers’ evaluative impressions. Motion Energy Analysis (MEA) has been suggested to solve this issue (Ramseyer and Tschacher, 2011). MEA quantifies general motor activity by calculating pixel changes between pre-filtered sequential video frames. More recently, the authors introduced a method to separate body and head movement within MEA (Ramseyer and Tschacher, 2014). MEA data, however, lack information about the form of movements and postures displayed. We suggest the use of motion capture technology to overcome this constraint. In contrast to MEA, motion capture technology issues detailed protocols of body movement including rotation and translation information for all joints (cf. Poppe et al., 2014; Cornejo et al., 2018). The rich data protocols resulting from motion capturing allow to analyze a broad variety of behavioral features as possible predictors of perceived rapport. These features include aggregates of movement activity across all body parts (comparable to MEA), as well as selections of specific nonverbal subsystems, such as gestures or head and body movements and postures. Most importantly, the synchronous movement protocols of both interaction partners allow one to establish dyadic coordination patterns in terms of temporal entrainment (synchrony) as well postural similarity (mimicry).

## Method: Observer Study

### Stimulus Material

Volunteer student participants were recruited for interaction recordings at the University of Cologne in Germany and at the American University of Sharjah in the United Arab Emirates (UAE). Recruitment in Sharjah focused on local Emiratis and students from surrounding Arab countries (Gulf States) whose mother tongue was Arabic. For the German portion of the sample, only native students were recruited in Cologne. Volunteers were randomly paired into homogenous female-female and male-male dyads. A major criterion was that the partners did not know each other before the sessions. If this was the case, the participants were reassigned to another pair. In total, 15 dyads were recorded in Germany and 15 in the UAE.

**FIGURE 1:**
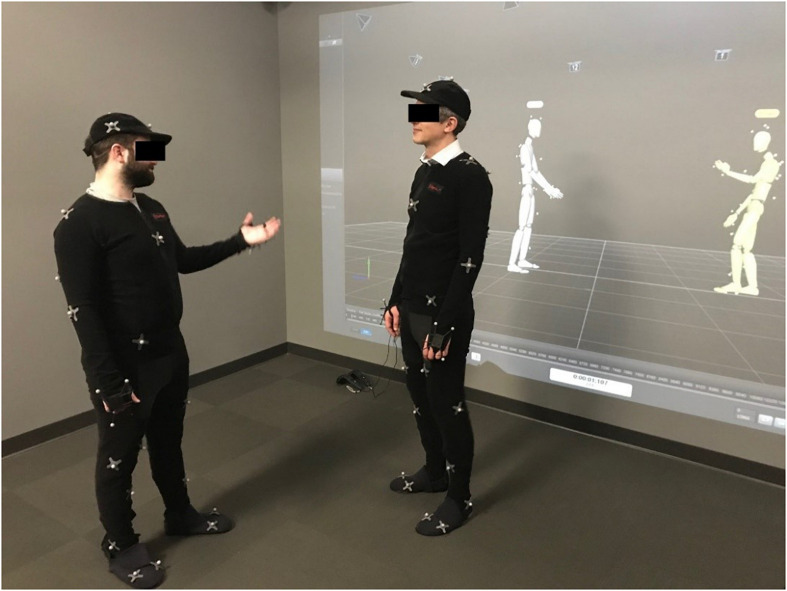
Posed interaction showing the setup and the interface of the capture software as projection in the background (180° rotated).

Participants were instructed that they would have a short 5–7 min conversation with another student during which they should get to know each other. Before the conversations began, participants were led into different rooms to put on the data suits necessary for motion capturing. A same sex student assistant placed the markers on the data suits and guided the interactors to the middle of the recording room where they met the experimenter. Motion capture was performed with a 12-camera Optitrack system and the capture software Arena (Optitrack, 2017). Cameras were positioned around a square area of 4 × 4 meters. Participants were then asked by the experimenter to take a T-pose (upright symmetric posture with legs closed and arms horizontally stretched out, palms down) for calibration of the tracking system. Then the participants were told that they could move freely in the square between the cameras and should use the next 5–7 min to get to know each other. Next, the experimenter left the room and the participants started the conversation. Using the capture software Arena, full body motion of both actors was captured during the conversation with a temporal resolution of 150 Hz. Figure 1 shows a dyad wearing the data suits with the IR reflecting markers and a projection of the capture software showing both virtual characters for demonstration purposes. After completing the interaction, the participants were debriefed and received 15 Euro (Cologne), or an equivalent on-campus restaurant voucher (Sharjah) for their participation.

**FIGURE 2:**
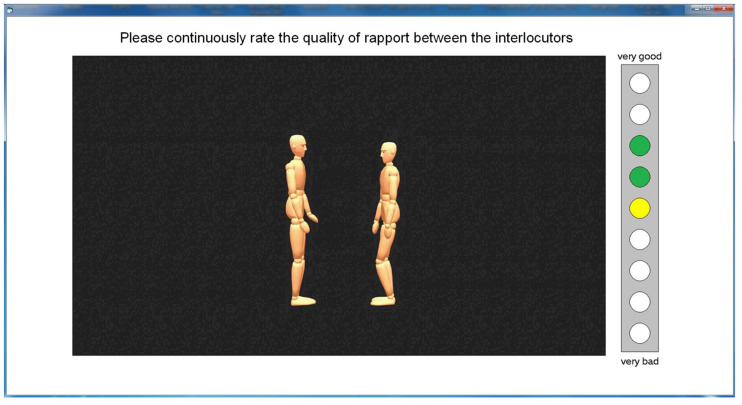
Screenshot of the user interface for the continuous judgment of rapport (description in the text).

Movement data of all dyads were transferred to the software MotionBuilder (Autodesk, 2017) for post-production to map the animation data to the neutral computer model and to handle and animation issues, such as jitter and penetrations of body parts. The computer model appeared as a wooden mannequin (cf. Bente et al., 2010) to standardize appearance and to obscure the gender and culture of participants (see Figure 2).

We selected five female and five male dyads from each country with the best recording quality (fewest recording errors, i.e., jitter, erroneous joint detection or dropouts and data drop-outs because of marker occlusions) as our final stimulus material. To provide comparable stimuli with regard to length we selected 1 min segments from the middle of all 20 interaction recordings. Using MotionBuilder, the stimulus sequences were then rendered to digital videos with a 25 frames/second frequency and in a 1,024 × 768 pixel resolution.

### Dependent Measures

The study focused on perceived rapport as the major variable. To account for potential variations in perceived rapport during the 1 min interactions, we used a real-time-response (RTR) measure (Bente et al., 2009) to indicate continuously the level of perceived rapport during observation. A 9-point rating scale with the extremes “+4” (very good rapport), “−4” (very bad rapport) and “0” (indifferent) was used for this purpose and was displayed as a gauge on the stimulus screen (see Figure 2).

As the computer characters were intended to obscure gender and culture of the stimulus dyads, we included two further questions at the end of each clip to check the effectiveness of this manipulation. We asked: (1) whether the participants assumed the respective dyad to be female or male and (2) whether they assumed the interactors to be of German or Arab origin. The respective hit rates should serve as a treatment check measure, to ensure that gender and culture were successfully obscured and perceived movement alone did not lead to the recognition of gender and culture and related stereotypes.

### Participants

Student participants for the perception study were recruited at the University of Cologne (Germany) and the American University of Sharjah (UAE) via local student mailing lists and seminar announcements aiming at an equal number of male and female observers. Twenty-six female and 24 male observers participated in the study in Cologne, and 25 female observers and 21 male observers participated in Sharjah. After analyzing the biographical data, we excluded eight participants from analysis who did not meet the selection criteria (i.e., born and raised either in Germany or an Arab country as well as having either German or Arab parents). The final data set consisted of 24 female and 23 male observers in Cologne (mean age = 24.87, *SD* = 6.66), and 22 female observers and 19 male observers in Sharjah (mean age = 20.22, *SD* = 2.33).

### Procedure

Up to six participants (between three and six depending on the number showing up) were seated in a large seminar room capable of holding 30 people, leaving at least six feet of distance between each of them. Each participant faced a laptop computer with a 15 (1,980 × 1,280) widescreen monitor. They were asked not to talk to each other during the experiment. Then they were shown a screen shot printout of the user interface of the experiment software (see Figure 2) and received the following instructions:

You will now see a series of short one-minute muted videos each showing computer animations of two people in a conversation. During the video you will be asked to provide your judgment about the dyad’s rapport; this means how well the two inter-actors are getting along with each other or are tuned-in during interaction. Please use the cursor up-down keys on your keyboard to continuously indicate your impression of their rapport. Your selections will be shown on the gauge on the right side of the screen (see picture in front of you). Moving the scale points on the gauge up into the green area means you have a positive impression of their rapport, moving down into the red area would indicate a negative impression. The more green or red dots light up, the better or worse is the dyads rapport, respectively. Your impression can change at any time during the interaction. Please use the cursors continuously to indicate any changes in your impression. After each clip you will be prompted, on a new screen, to indicate your opinion on whether this dyad was a female or a male dyad and whether the dyad portrayed Germans or Arabs. If you have any questions you can ask now. If you are ready, please hit the start button on the screen to launch the experiment.

Participants then started the video sequences that were presented in random order. Figure 2 shows the screen layout during the stimulus presentation with the RTR gauge displayed at the right of the video window. The RTR gauge could be controlled by pressing the cursor-up or cursor-down keys on the computer keyboard. At the end of the video, a new screen appeared asking for the dyads’ gender (male or female), followed by a screen asking for the dyads’ culture of origin (German or Arab).

## Results: Observer Study

### Control Check

To ensure the efficiency of our stimulus manipulation in masking stimulus gender and culture, we tested whether recognition rates for gender and culture were significantly different from chance level. Two separate one-sample *t*-tests were conducted for both variables using the chance level of 10 hits out of 20 dyads as criteria. Results indicated no significant difference from the chance level for either gender or culture. Mean hit rates were *M* = 10.30, *SD* = 2.64, *t*(87) = 1.05, *p* = 0.30 for gender, and *M* = 9.97, *SD* = 2.27, *t*(87) = −0.14, *p* = 0.89, for culture. This indicated that the participants were not able to identify reliably the gender or culture of the avatar dyads from their appearance nor from their nonverbal behavior. Accordingly, we excluded stereotype influences as accounting for variance in the rapport judgments.

### Observer Agreement

To test whether perceptions of nonverbal rapport are consistent within and across the observer groups we first conducted intra-class correlations for each observer group (female German, male German; female Arab, male Arab) based on the rapport ratings toward the whole stimulus set. The correlations are presented in Table 1.

**TABLE 1:** Intraclass correlations for average rapport ratings across all stimuli.

Culture	Gender	ICC	df	*F*
German	Female	0.757***	25, 475	4.113
	Male	0.577***	23, 437	2.365
Arab	Female	0.595***	24, 456	2.471
	Male	0.566***	20, 380	2.304

***p < 0.001.

**FIGURE 3:**
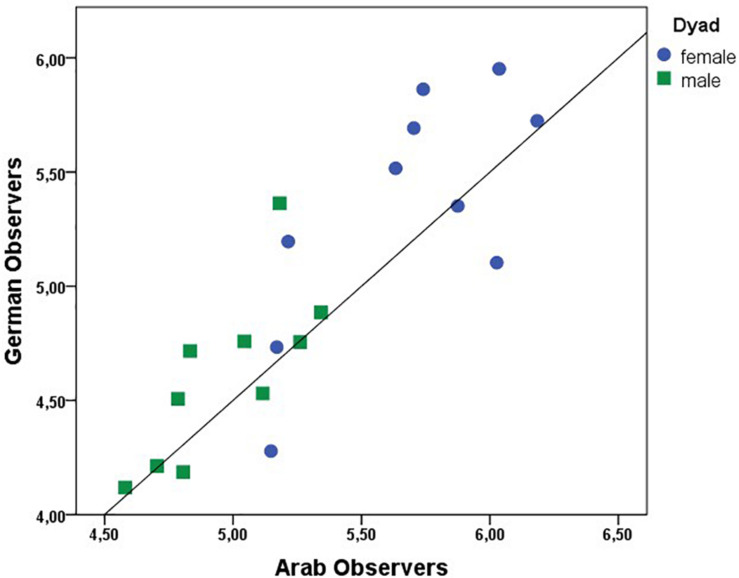
Correlation graph for German and Arab observers, differentiated for male (green squares) and female (blue circles) stimulus dyads.

As Table 1 shows, there was high agreement within observer groups in terms of rapport ratings toward all stimuli. Next, we aggregated the rapport ratings for the four groups of observers, generating a data matrix that contained the stimuli as cases and the average ratings of each group as variables. We then ran Pearson-Product-Moment-Correlations for the four observer groups across the 20 stimuli. The correlations are displayed numerically in Table 2, as well as visually in Figure 3. All groups significantly correlated in their rapport ratings, with correlations explaining between 57 and 77% of the variance. Figure 3 illustrates that the correlations were not merely driven by the gender differences but also reflect correlation within the stimulus categories.

**TABLE 2:** Pearson’s product moment correlations for average rapport ratings across 20 dyads.

Observers	Female German	Male German	Female Arab	Male Arab
Female German		0.877***	0.785***	0.755***
Male German			0.792***	0.772***
Female Arab				0.805***
Male Arab				

***p < 0.001; N = 20 for all analyses.

### Dynamics of Rapport Ratings

**FIGURE 4:**
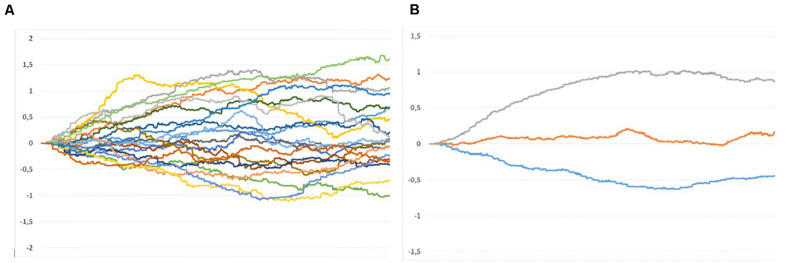
Continuous RTR rapport ratings. **(A)** Averaged for each of the 20 stimulus clips across 88 observers. **(B)** Averaged for 7 high, 6 neutral, and 7 low rapport dyads.

Regarding the dynamics of the continuous rapport ratings, we conducted an exploratory graphical analysis of the RTR process measures. For this purpose, we averaged the RTR data across observers over time for each of the 20 dyads. Figure 4A shows the resulting time graphs. It illustrates that with only a few exceptions, average rapport ratings show little spontaneous fluctuations or any significant changes in direction during the 1 min sequences. Rather, the curves suggest that observers very early (after about 5 s) take a certain judgment direction and asymptotically approach a relatively stable level after about 30 s. This tendency becomes even more evident when averaging the dyads with high, medium, and low average rapport ratings. Figure 4B shows the averaged curves for the seven clips with the highest, the seven with the lowest, and the six with medium rapport ratings (lying between the seven high and low scoring stimuli). It supports the assumption that there is an initial judgment tendency driven by thin slices of behavior and that further observations just serve to consolidate the swift first impressions. It remains an open question, though, whether this process is driven by consistent stimulus characteristics, aggregating in the observers’ impressions over time, or caused by selective perceptual strategies of the observers, who rapidly form their impression and then assimilate further observations.

### Gender and Cultural Differences

To analyze the effects of the observers’ and the dyads’ gender and culture on perceived rapport we conducted a 2 × 2 × 2 × 2 mixed ANOVA including culture of observers (German vs. Arab) and gender of observers (female vs. male) as between-subject factors and the culture of dyad (German vs. Arab) and gender of dyad (male vs. female) as within-subject factors. Average rapport ratings for the 1 min sequences served as the dependent variable. Between-subject factors were included despite the high inter-observer correlations to identify potential interaction effects due to in-group familiarities.

We found a main effect for the culture of observers, *F*(1, 87) = 12.96, *p <* 0.001, η^2^*_*p*_* = 0.134, but no main effect for gender of observers or any interaction effect between observer factors and stimulus factors. The main effect shows that Arab observers (*M* = 5.29, *SD* = 0.38) were, in general, more positive in their rapport ratings than German observers (*M* = 4.99, *SD* = 0.40). It remains unclear whether this finding reflects a general positivity bias in the social judgments of Arabs as compared to Germans, or a different sensitivity to rapport cues. As there were no interaction effects between the culture of observers and any other factor, we refrained from correcting the bias by grand mean standardization (Fischer, 2004).

**FIGURE 5:**
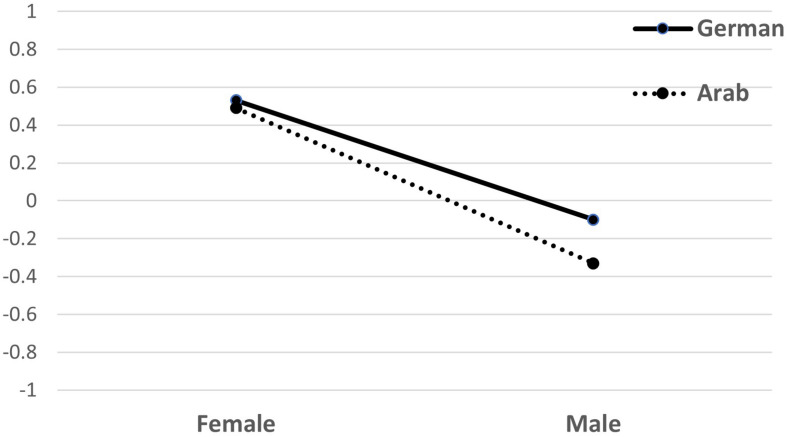
Interaction graph for rapport ratings (estimated marginal means) depending on gender and culture of the stimulus dyads. The *y*-axis scale is set from –1 to +1 here to showcase the effect (the participants rated from –4 to +4).

We further found a significant main effect for the dyads’ gender, *F*(1, 87) = 149.99, *p <* 0.001, η^2^*_*p*_* = 0.641, indicating that the female dyads (*M* = 5.50, *SD* = 0.49) were perceived as doing better in nonverbal rapport building than male dyads (*M* = 4.78, *SD* = 0.52). We also found a main effect for the culture of the dyads, *F*(1, 87) = 6.62, *p* = 0.012, η^2^*_*p*_* = 0.073 indicating that German dyads were perceived higher in rapport than the Arab dyads. This main effect is, however, explained by an interaction between the gender and culture of the dyads, *F*(1, 87) = 4.0, *p* = 0.049, η^2^*_*p*_* = 0.045. Only the male Arab dyads were rated lower in rapport than male German dyads, *t*(87) = 2.87, *p* = 0.005, *d* = 0.37, while female dyads showed no difference between the two cultures, *t*(87) = −0.75, *p* = 0.391, *d* = 0.10. Figure 5 illustrates the interaction effect.

## Method: Behavior Analysis

### Rationale

The behavioral analyses aim to showcase the information richness of the motion capture data and to demonstrate multiple ways to extract and examine the nonverbal interaction patterns that underlie the perception of dyadic rapport. We here focus on a set of behaviors that have been described in the literature as relevant to rapport. These are: *expressivity, mutual attention, reciprocal positivity*, and *coordination* (Tickle-Degnen and Rosenthal, 1990; Bernieri et al., 1996; Grahe and Bernieri, 1999; Tickle-Degnen, 2006), the latter including aspects of *synchrony* (i.e., the temporal entrainment of interactors’ movement activity; Lakin and Chartrand, 2003; Lakens and Stel, 2011; Ramseyer and Tschacher, 2011; Fujiwara and Daibo, 2016) and *mimicry* (i.e., the similarity of movements and postures in form; Bernieri et al., 1994; Lakin and Chartrand, 2003; Miles et al., 2009). Importantly, we here conceive coordination as orthogonal to the individual behaviors that are subject to coordination and that have to be further processed to reveal the respective dyadic interdependencies. We exemplarily selected the following individual behaviors: (1) rotational head orientation as a proxy for visual attention (Loomis et al., 2008), (2) approach/distancing movements as an indicator of positivity/negativity (Sundstrom and Altman, 1976), and (3) overall movement activity (i.e., aggregate positional changes of all joints) as a measure of expressivity, comparable to MEA (Nelson et al., 2016). These behaviors were further submitted to algorithms quantifying the behavior of the dyads as a whole and the respective spatial–temporal coordination patterns.

### Feature Extraction

A Python plugin for MotionBuilder was developed to export global translation of 15 body joints for each interaction partner from the .FBX (filmbox format) animation files to .CSV files. We also exported the coordinates for three additional virtual markers attached to the nose and the ears of the interactors. These coordinates were used to calculate head rotation instead of using the Euler angles in the .FBX files, which are difficult to interpret. Translation data were exported as absolute metric values in the shared 3D world coordinate system. Further calculations were based on these data sets (see Leuschner, 2013). During export from the capture system to MotionBuilder data, a different scale factor was used for all the Arabic dyads and one German dyad. The scale factor deviated from the real world dimensions by the factor 5/6. All sizes and distances for those data sets were therefore corrected, i.e., upscaled by the factor 1.2 before being used in parameter formation and statistical analysis.

**FIGURE 6:**
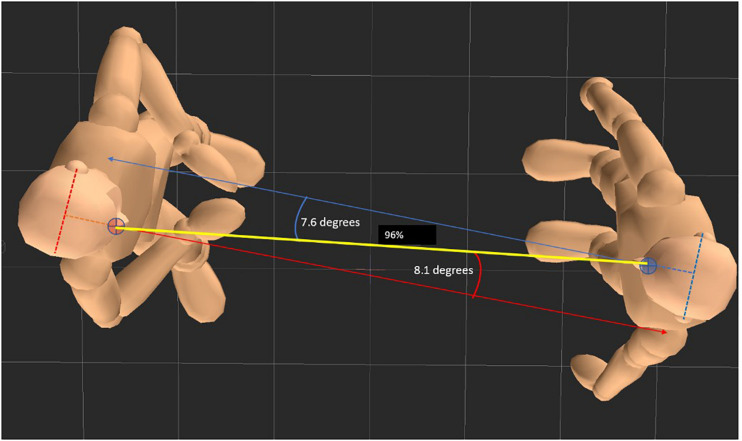
Rationale for measuring rotational head orientation and interpersonal proximity. Invisible virtual markers between the characters’ eyes were used as anchor for distance measurement and as references for the assessment of rotational deviations from the direct line of view. The angle was calculated as orthogonal to the line between two virtual ear markers projected onto the x/z plane. Distance was calibrated as percentage of both partners’ arm lengths.

To cover individual behaviors relevant to orientation, distance, and activity we extracted three behavior vectors for each interaction partner from the data matrices:

(1) Orientation: We calculated the angular deviation of the individual head rotation from the direct line of view, which was defined as the dynamically changing line between both computer models’ nose markers (see Figure 6).
(2) Distance: We calculated movements toward or away from the partner as consecutive Euclidian distances between the position of the nose marker of one partner at timepoint t and the position of the nose marker of the other person at timepoint t − 1 (see Figure 6).
(3) Activity: We aggregated the positional changes of all 15 joints between consecutive time points (position changes were z-transformed for each joint separately before aggregation).

**FIGURE 7:**
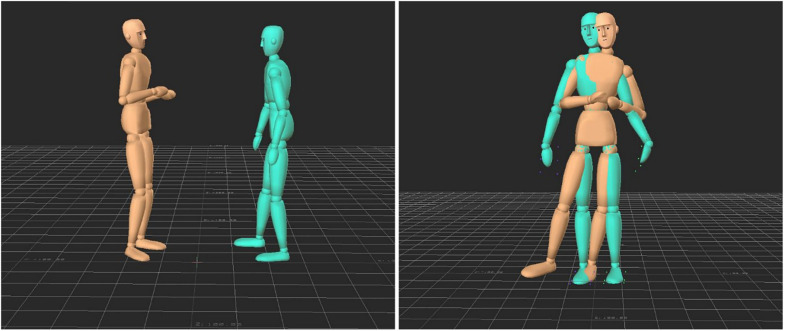
Rationale for multidimensional comparisons: Both skeletons’ hips were constrained to the position as well as the y-rotation of a static object in the coordinate system origin, thus always pointing to the front. Postural differences were calculated as Euclidian distances between the corresponding joints of both partners for each timepoint.

To enable multidimensional comparisons of postural similarity (mimicry) we further extracted translation matrices from the animation files of both partners containing the 3D coordinates of the 15 body joints. Following the procedures suggested by Poppe et al. (2014) positional data of the joints were normalized to compensate for different body sizes and the skeletons of both partners were snapped to the shared coordinate system’s origin and y-rotations were frozen. Both hips thus were always fixed in the same position and oriented toward the front of the scene. The postural similarity was then calculated for each point of time as the sum of distances between the interaction partners corresponding joints. The rationale of the procedure is illustrated in Figure 7. The transformations as well as the CSV-export of the transformed data were performed with the MotionBuilder Python plugin.

### Parameter Formation

The extracted vectors/matrices were further processed in two ways. First, we calculated compound measures to characterize the dyads’ nonverbal behavior as a whole. These measures comprised: (1) orientation: the mean absolute deviation from the direct line of view averaged for both partners and the percentage of time both partners’ head deviation was less than 10° from the direct line of view (see Figure 6), (2) distance: the average of the Euclidian distance between the partners nose markers over time, and (3) activity: the mean of aggregated position changes across all 15 joints and both partners, and the percent of time of simultaneous activity and inactivity of both partners.

Second, the individual vectors were submitted to a suite of algorithms to quantify different aspects of coordination (see Cheong, 2019 for the algorithms and Python codes). We applied: (1) Pearson correlations, (2), Mutual Information (MI), (3) Rolling Window Time Lagged Cross-Correlation (RWTLCC), and (4) Dynamic Time Warping (DTW). DTW has only recently been introduced to the field of gesture analysis (Ten Holt et al., 2007; Chen et al., 2019). A major advantage of the method is that it takes into regard differences in form and time when comparing two signals. We also applied DTW to the multidimensional translation matrices that were extracted from the .FBX animation files after snapping the hips of the two partners (see Figure 7). Instead of the point-to-point differences between two vectors we here used the local sum of the point to point Euclidian distances of all 15 joints to fill the DTW data matrix. These data were also used to calculate the mean postural difference over time.

For RWTLCC we applied a step-size of 0.2 s (5 datapoints), a lag of ±2 s (±50 datapoints) and a rolling window size of 5 s (125 datapoints). To quantify the mutual interdependencies of the two signals we calculated the absolute offset of the correlation peak from the zero lag as well as the average maximal correlation found at this point. Different filters were used for the three behavioral dimensions using the “scipy.signal” library. Head rotation angles were lowpass filtered with a constant of 0.25 and standardized (divided by standard deviation) keeping the dynamic zero point as “straight orientation toward the partner.” This allowed us to focus on more stable orientation patterns instead of rapid local movements (e.g., head shakes). Approach and distancing movements as well as overall motion were lowpass filtered with a filter constant of 1.0 to suppress observable jitter in the data.

Pearson “r” and the entropy measure resulting from MI analysis were used as input for statistical analysis. FromRWTLCC we calculated the average maximal correlation at each point across all time lags as well the absolute offset of the correlation peak from the zero lag as general coherence and synchrony indicators. We further used the “DTW distance” measure, i.e., the minimum path cost (Cheong, 2019), to quantify the (dis)similarity between the behavioral vectors of the interactants for further analyses.

## Results: Behavior Analysis

Pearson correlations were conducted for the *N* = 20 dyads between the extracted behavioral parameters and the average rapport ratings they received. The results for the aggregate dyadic measures are shown in Table 3. A significant negative correlation was found between the interpersonal distance of the partners and the rapport ratings, indicating that a closer stance was associated with higher levels of perceived rapport. Neither one of the aggregate orientation or activity parameters showed a significant correlation with rapport.

**TABLE 3:** Correlations between rapport ratings and the dyadic aggregate measures.

	Parameters	Correlation
Orientation	Average degree of head deviation	–0.093
	% of time head deviation < 10°	–0.265
Distance	Average distance between nose markers	−0.489*
Activity	Average degree of movement	0.360
	Both partners in motion	0.343
	Both partners inactive	–0.400

*p < 0.05; N = 20 for all analyses.

**FIGURE 8:**
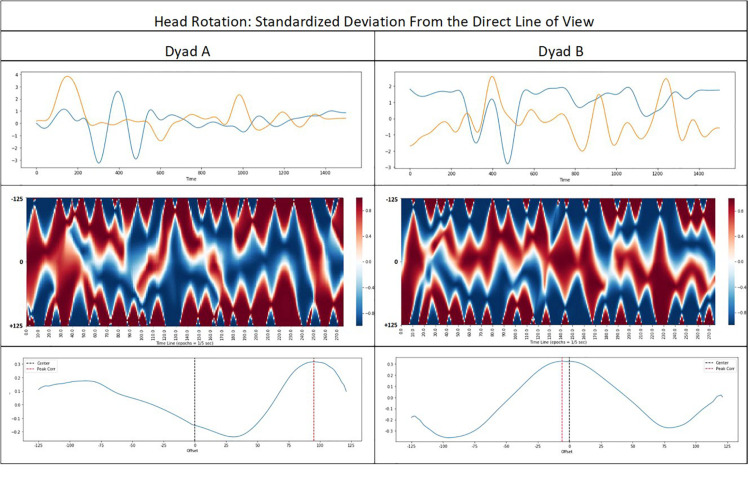
Results of RWTLCC for the head rotation dynamics of 2 dyads differing in the peak correlation offset. The upper graphs show the low pass filtered, standardized head rotations. The mid graphs show the time lagged correlations with lags of ±5 s (±125 data points) and a moving window with a step size of 0.2 s (5 data points). The lower graphs show the mean correlations for the time lags from –125 to +125 datapoints.

Coordination analysis of individual parameters showed a different picture. Results are summarized in Table 4. For the rotational head movements (i.e., turning away from or toward the partner’s actual position as a proxy for directed gaze and visual attention), Pearson correlations as well as peak correlations in the ± 5 second window of RWTLCC showed significant negative correlations with rapport ratings: the lower the peak correlation between the orientation parameter, the higher were the rapport ratings. This suggests that rotational head movements in high rapport dyads occur in a complementary rather than in a symmetric fashion. Figure 8 illustrates the result for two typical dyads. In contrast to the head rotation we found positive correlations of perceived rapport and interpersonal distance variations. The higher the peak correlation in distancing and approach behaviors within the ± 5 second window (the better coordinated the moves toward or away from each other), the higher was the perceived rapport level. Similarly, we found a significant negative correlation for the offset of the peak correlations between the two motion vectors (aggregated Euclidian distances between 15 joints) and the rapport ratings. The smaller the offset for a correlation peak in the lag window, the higher were the rapport ratings, which seems to corroborate findings from prior MEA studies (Ramseyer and Tschacher, 2011, 2014) suggesting a correlation between motor synchrony and rapport. DTW measures did not correlate in any of the parameters.

**TABLE 4:** Correlations between rapport ratings and coordination parameters for the individual behavior vectors.

	Coordination parameters				

	Pearson r	Mutual Information	RWTLCC: peak correlation	RWTLCC: peak offset	DTW: distance
Orientation	−0.499*	0.006	−0.447*	0.340	0.218
Distance	–0.140	–0.014	0.545*	–0.432	–0.030
Activity	–0.321	–0.421	0.020	−0.533*	–0.401

*p < 0.05; N = 20 for all analyses.

Correlations between rapport ratings and the multidimensional measures of postural similarity (mimicry), based on the aggregated Euclidian differences of the 15 joints, did not reach significance level. The similarity measure resulting from multidimensional DTW correlated with rapport ratings at *r* = 0.359 (*p* = 0.120). A similar correlation was found for the postural similarity measure, i.e., the larger the average joint distances (dissimilarity), the higher were the rapport ratings (*r* = 0.367, *p* = 0.111). It is worth noting, that the “minimal warp path length” and “average joint distance” correlated highly with one another (*r* = 0.993, *p* = 0.001). Average distance calculations are much faster to compute than DTW. It remains to be tested with larger samples whether both algorithms are functionally equivalent which would have important implications for their application in future studies.

## Discussion

The presented work addressed two methodological problems pertinent to the analysis of nonverbal rapport: (1) the production of standardized stimuli that allow for unbiased observer judgments and (2) the assessment of nonverbal interaction patterns that drive these judgments. We suggested motion capture and character animation as possible solutions (cf. Cornejo et al., 2018; Bente, 2019). We applied this methodology exemplarily to the study of gender and cultural differences in the perception of rapport as both person characteristics can be discerned from video and are prone to elicit stereotypes relevant to the judgment of rapport. Rapport is defined as a phenomenon only observable in interactions (Tickle-Degnen and Rosenthal, 1990), specifically depending on the coordination of nonverbal activity. In this sense, efforts to identify its behavioral underpinnings challenge the quality of the dyadic interaction data and the availability of algorithms to quantify the interpersonal coordination patterns (Bente and Novotny, 2020). We aimed to show that motion capture data provide unique possibilities to encounter this challenge.

Aiming at a maximal contrast between the groups in the first study of this kind, we focused on homogenous female and male dyads and selected German participants as representatives of a Western, individualistic and more emancipated culture and Middle East Arab participants (born in the United Arab Emirates or a Gulf State) to represent a more collectivistic culture with a more traditional gender role conception. Observers were also recruited from both regions. The results strongly support the novel approach. Using avatar animations instead of video we were able to successfully mask the gender and culture of the stimulus dyads. Recognition rates for both variables did not significantly deviate from chance level. On the other hand, rapport ratings correlated highly within and across observer groups (female/male, German/Arab) pointing to universal, gender and culture independent judgment policies (Bernieri and Gillis, 1995a). The significant inter-observer correlations also indicate that rapport can be reliably judged exclusively from the movement activity in a dyadic interaction (cf. Grahe and Bernieri, 1999) and that the nonverbal cues relevant to impression formation can be effectively portrayed by the avatar animations (Bente et al., 2010).

Beyond the high correlations in the rapport judgments, observer ratings also showed an interesting temporal dynamic. Using for the first time a continuous rating technique for rapport, we found that individual ratings already start to converge after a few seconds of observation and then asymptotically approach a final and robust level after about 20 s of the 1 min sequences. Our results corroborate earlier findings showing that rapport can be judged from a few seconds of interaction behavior (Grahe and Bernieri, 1999) and are also consistent with a “thin slices” perspective on the perception of nonverbal behavior in general (Ambady and Rosenthal, 1992). The result suggests that rapport impressions are formed swiftly and that further observations are used to consolidate the first impression rather than being sensitive to spontaneous fluctuations. In fact, this can be an effect of a perceptual bias, primed by the first impression, as well as a sign of the consistency of the specific rapport level exerted by the dyads. To test whether continuous ratings are sensitive toward local changes in nonverbal rapport one could apply the “pseudo interaction paradigm” (cf. Bernieri et al., 1988; Ramseyer and Tschacher, 2010) combining interactors from different dyads, parts of different interactions, or natural and synthetic behaviors. For instance, segments of high rapport dyads could be concatenated with segments of low rapport dyads or randomly assembled interactors from different dyads. Animation tools such as MotionBuilder provide powerful solutions to create such pseudo dyads and to smoothen the transitions by interpolating potentially distant joint postures at the seams of two segments.

An ANOVA conducted across gender and culture of stimuli and observers revealed no effect of observer gender on the general level of rapport ratings, nor did we find any interaction effects between observer gender and the other group variables (i.e., observer culture, stimulus gender, or stimulus culture). These results stand in contrast to the findings of Puccinelli et al. (2003), who reported a general positivity bias of female observers being more accommodating (i.e., generally ascribing higher rapport levels to the observed dyads). The gender effect reported by these authors, however, might be because the nearly exclusively female stimulus dyads distinctively primed rapport relevant self stereotypes in female observers. This explanation receives some support from the current results as obscuring the gender of the stimuli completely eliminated gender effects on the observer side. Future research should continue investigating this interesting interaction effect.

Regarding observer culture, we found a main effect wherein the Arab observers rated stimuli as generally higher in rapport than did the German observers. This could reflect either a general positivity bias or a different sensitivity to rapport cues. The latter assumption would be consistent with findings revealing cultural differences in nonverbal behavior between Arabs and another Western culture (i.e., US Americans; Watson, 1970). Specifically, Arabs are thought to be a higher “contact” culture, utilizing more touch and direct gaze cues, compared to their Western counterparts. As Bernieri and Gillis (1995a) note, “One might expect greater attention to such behaviors by Arabs assessing rapport than would Americans” (p. 117). It is possible that Arabs in our study were more attentive to these contact cues than Germans and thus rated stimuli higher in rapport across the board. The high correlations between Arab and German ratings, however, indicate that this difference might only concern a shift of the scale mean whereas relative perceptions of dyadic rapport are well aligned across cultures.

Beyond the intended proof of concept for the novel methodology the ANOVA results also stimulate further theoretical thoughts regarding the role of gender and culture in the establishment and maintenance of rapport. Unanimously, female dyads from both cultures were judged as significantly higher in rapport than male dyads. The perceived difference between female and male dyads was even more accentuated within the Arab dyads as revealed by a significant interaction effect. German males were rated as higher in rapport than Arab males whereas German and Arab females were rated equally in rapport. When juxtaposed with the main effect of dyad gender these findings suggest that the extent of perceived gender differs between cultures. This is consistent with the general insight that culture plays a crucial role in the establishment of gender-role expectations, respective socialization practices and resulting social behaviors (Williams and Best, 1994; Van de Vijver, 2007). As Hall and Briton (1993) posit: “Pressure to conform to stereotypes can be great. Men who are gesturally or facially expressive, for example, may be stigmatized as being weak or feminine…” (p. 283). Expressivity has been identified as a crucial behavioral feature in the perception of rapport (Bernieri and Gillis, 1995a) and as the “raw action material” in nonverbal coordination (Tickle-Degnen, 2006, p. 387). A medium level of expressivity appears to be ideal for the establishment of rapport. Nelson et al. (2016) hold that “According to Tickle-Degnen’s (2006) model, optimal experiences of rapport are those where dyads feel and act in calm, yet attentive ways; suboptimal experiences foster overactive or underactive levels of action and affect. More specifically, when an actor’s expressivity is overactive, information is lost between an actor and a perceiver. When expressivity is underactive, there is a shortage of nonverbal information passed between partners” (p. 3). Conceding that the Arab culture in our study has more fixed roles of masculinity and femininity than the German culture, a use of less expressive behavior by the Arab males might explain their garnering of comparably low rapport ratings.

Given the small stimulus sample size in our study, interpretations regarding gender and cultural factors in the generation of rapport should be treated with caution. Yet, our observations can be taken as a starting point for future research that investigates rapport building behavior across gender and cultures more directly. On the one hand, studies of this kind would have to be based on significantly larger interaction samples. On the other hand, they would require a theoretical framework that allows one to conceptualize the influence of gender and culture as well as their interplay in stimulating relational orientation and fostering rapport relevant behaviors. As suggested by Cross and Madson (1997) the construct of “self construal” (Markus and Kitayama, 1991) could provide such a framework. Central to the construct is the distinction between interdependent and interdependent self construals (Markus and Kitayama, 1991). This distinction refers to the way people see their self either as integral part of the group emphasizing harmony and unity or as an isolated entity striving for uniqueness and individual achievement. The construct has been primarily applied to cultural differences (Kitayama et al., 2007; Harb and Smith, 2008; Gore et al., 2009; Cross et al., 2011). Yet, a few studies also successfully applied it to the understanding of gender differences (Cross and Madson, 1997; Watkins et al., 2003). For instance, Cross and Madson (1997) found that the cultural environment even within a Western civilization still fosters independence and autonomy in men and interdependence and relatedness in women. They further hypothesize that such differences in the individuals’ cognitive structure affect the micro-level of social interactions in the sense that “… individuals with an interdependent self-construal may develop skills and behaviors that facilitate the development of close relationships with others” (p. 17). While self construal research has predominantly focused on cognitive variables (e.g., Cross et al., 2002; Konrath et al., 2009), little is known about its influence on how people concretely establish rapport on the micro level of social interactions. We contend, that our understanding of the cognitive and behavioral mechanisms that foster rapport and enable or disable a smooth flow of communication could largely benefit from studies combining the assessment of self construals (cf., Singelis, 1994) with the introduced methods to analyze perceptions and behavioral correlates of rapport.

The explorative behavior analyses demonstrated the manifold possibilities to extract nonverbal features from the motion capture data and to quantify respective dyadic coordination patterns. Some of the tentative results might also inspire future studies beyond mere method demonstration. For instance, RWTLCC revealed some interesting correlations with rapport ratings. The mean peak correlation of the head rotation behavior of both partners (i.e., the highest correlation across the time lags (±2 s) found for each data point averaged over the whole 1 min sequence) correlated negatively with perceived rapport. This indicates, that the more similar the orientation dynamics of both partners within a critical time window was (i.e., both turning way from or toward the partner), the lower was the level of perceived rapport. However, the same RWTLCC parameter (mean peak correlation) for the behavioral dimension “approach and distancing behavior” of the interactors was positively correlated with rapport (i.e., the more similar the distancing and approach motions, the higher the rapport ratings). A further significant result occurred for the entrainment of the general movement activity. Here we found a negative correlation between the average offset of the peak correlation and the rapport ratings, indicating that the closer the correlation peaks to the zero-lag point (simultaneity), the higher were the rapport ratings. Overall, these results shed some critical light on previous conceptions of the role of synchrony and mimicry for rapport building (Chartrand and Bargh, 1999; Lakin and Chartrand, 2003; Miles et al., 2009; Lakens and Stel, 2011), and point to the necessity to treat the various behavioral subsystems differently with regard to the type and the level of coordination that is functional for rapport.

## Limitations and Future Prospects

The major limitation of the current study is the small number of interaction stimuli that only consisted of 20 dyads with 5 dyads for each gender and culture combination. It is important to reiterate, though, that the primary purpose of this study was to demonstrate the benefits of the new methodology. The possibilities of the proposed methodology reach far beyond the study of rapport and gender and cultural differences. In fact, the elimination of stereotype influence on the observer side as well as the access to objective behavioral measures provide a more universal solution for observation studies in nonverbal research, including traditional domains, such as impression formation, person perception, deception, and emotion recognition. The quality of the obtained data sets would ideally fill both sides of prominent effect models in nonverbal communication research such as Brunswik’s lens model (Brunswik, 1956; Bernieri et al., 1996). Future theory driven studies following this approach certainly would require larger stimulus data sets.

This relates to a further limitation of the study. It concerns the use of a marker-based motion capture device that requires laboratory setup and larger amounts of time to equip and calibrate the participants. At this time, motion capturing still provides the most accurate method to overcome the described measurement issues in nonverbal behavior research and the fact that such systems are now rather affordable and easy to use is likely going to facilitate more widespread adoption in research and practice. However, broadly accessible machine learning tools can be expected to replace motion capture in the near future, allowing one to extract skeletal motion data from standard video (Cao et al., 2018). As the resulting data protocols (joint translations and rotations) will be compatible with motion capture data, parameters and analysis tools developed and applied to motion capture data will retain their validity.

A third limitation concerns the selective choice of behavioral parameters and algorithms to quantify patterns of coordination across different nonverbal subsystems. For demonstration purposes we here focused on a subset of the manifold possibilities to quantify behavioral interdependencies in interactions. Further algorithms and software tools can be found in Delaherche et al. (2012) and Varni et al. (2015). Promising approaches to analyze the temporal entrainment of two behavioral vectors in the frequency domain have also been introduced recently using Cross-Wavelet-Transform (Fujiwara and Daibo, 2016). It is important to note that the different algorithms are not just different ways to capture the same phenomenon, but distinctly define what is conceived as coordination or synchrony (Novotny, 2020). Therefore, further studies are needed to comparatively evaluate the different approaches with regard to their effectiveness in predicting the subjective experience or perceptions of rapport in varying contexts (cf. Bente and Novotny, 2020).

Lastly, the current study shows a limitation with regard to the measurement of perceived rapport. One might argue that it would be more important to assess the interactors’ experience of rapport rather than the impressions of neutral observers. In fact, there might be fundamental differences between a first-person perspective and a third person perspective on dyadic rapport (Bernieri and Gillis, 1995a, cf. Schilbach et al., 2013). The current study focused on the third person perspective as the major objective was to demonstrate the potential of the novel methods to eliminate stereotype effects and judgment biases specifically in observer studies. It is an interesting question for future research though, why self-reports and observer judgments drift apart. Bernieri and Gillis (1995a) supposed that this might be due to the fact observers refer to socially appealing cues (such as smiles) that are less relevant for rapport and oversee more relevant ones (such as nonverbal coordination). In the current study, facial expressions were not shown in the character animation stimuli and observers could base their judgments solely on the dyadic movement patterns. Whether this might have led to a higher agreement with the interactors’ self-rating remains an open question to be answered in upcoming studies. Another reason for discrepancies in rapport judgments might be that observers see the dyad as a whole whereas interactors see it from the individual perspective. Observers thus might be closer to the reality of the emergent dyadic construct of rapport than the interactors, whose evaluations can be influenced by other components of person perception such as for instance liking and control (cf. Human et al., 2013). This implies that interactors can also show discrepancies in their rapport judgments, which makes it generally questionable how these impressions can be used to validate observer judgments. Against this background it might make more sense to treat first and third person judgments of rapport as distinct, yet both relevant, information. It is important to note that impression management is not limited to preserve the individual’s face but also to create a positive impression of the group as a whole in front of an “audience” (cf. Goffman, 1959; Manning, 2005; Picone, 2015). The third person perspective thus might add relevant data for our understanding of nonverbal rapport mechanisms.

## Data Availability Statement

Raw data and stimulus materials developed in this project will be made available by the authors, without undue reservation, to any qualified request.

## Ethics Statement

The studies involving human participants were reviewed and approved by IRB of the American University of Sharjah (UAE) and the Department of Psychology at the University of Cologne (Germany).

## Author Contributions

GB and AA developed the study design and organized the data collection in the UAE and Germany. GB and DR conducted the observation studies in both countries. GB and EN analyzed the behavioral data and wrote the first manuscript draft. All authors contributed to the article and approved the submitted version.

## Conflict of Interest

The authors declare that the research was conducted in the absence of any commercial or financial relationships that could be construed as a potential conflict of interest.
