# Corrigendum: Beyond Stereotypes: Analyzing Gender and Cultural Differences in Nonverbal Rapport

**DOI:** 10.3389/fpsyg.2021.675148

**Published:** 2021-04-15

**Authors:** Gary Bente, Eric Novotny, Daniel Roth, Ahmad Al-Issa

**Affiliations:** ^1^Department of Communication, Michigan State University, East Lansing, MI, United States; ^2^Grady College of Journalism and Mass Communication, University of Georgia, Athens, GA, United States; ^3^Department of Computer Aided Medical Procedures and Augmented Reality, Technical University of Munich, Munich, Germany; ^4^Department of English, American University of Sharjah, Sharjah, United Arab Emirates

**Keywords:** rapport, nonverbal behavior, motion capture, character animation, gender, culture

In the original article, there was an error.

In section *Method: Behavior Analysis, Parameter Formation, Paragraph 4*, we said: “From DTW we calculated the length of the optimal warp path as a measure of similarity of the behavior vectors when stretched or compressed in time.” This is incorrect. We did not use “path length” but “DTW distance” for further statistical analyses.

A correction has been made to the section *Method: Behavior Analysis, Parameter Formation, Paragraph 4*. The corrected paragraph is shown below.

Pearson “r” and the entropy measure resulting from MI analysis were used as input for statistical analysis. From RWTLCC we calculated the average maximal correlation at each point across all time lags as well the absolute offset of the correlation peak from the zero lag as general coherence and synchrony indicators. We further used the “DTW distance” measure, i.e., the minimum path cost (Cheong, 2019), to quantify the (dis)similarity between the behavioral vectors of the interactants for further analyses.

Additionally, there was also an error in *Method: Observer Study, Stimulus Material, Paragraph 2*. In the phrase “Cameras were around a square area of 4 ×4 meters,” the word “positioned” is missing.

A correction has been made to the section *Method: Observer Study, Stimulus Material, Paragraph 2*. The corrected paragraph is shown below.

Participants were instructed that they would have a short 5–7 min conversation with another student during which they should get to know each other. Before the conversations began, participants were led into different rooms to put on the datasuits necessary for motion capturing. A same sex student assistant placed the markers on the data suits and guided the interactors to the middle of the recording room where they met the experimenter. Motion capture was performed with a 12-camera Optitrack system and the capture software Arena (Optitrack, 2017). Cameras were positioned around a square area of 4 ×4 meters. Participants were then asked by the experimenter to take a T-pose (upright symmetric posture with legs closed and arms horizontally stretched out, palms down) for calibration of the tracking system. Then the participants were told that they could move freely in the square between the cameras and should use the next 5–7 min to get to know each other. Next, the experimenter left the room and the participants started the conversation. Using the capture software Arena, full body motion of both actors was captured during the conversation with a temporal resolution of 150 Hz. Figure 1 shows a dyad wearing the data-suits with the IR reflecting markers and a projection of the capture software showing both virtual characters for demonstration purposes. After completing the interaction, the participants were debriefed and received 15 Euro (Cologne), or an equivalent on-campus restaurant voucher (Sharjah) for their participation.

The authors apologize for these errors and state that they do not change the scientific conclusions of the article in any way. The original article has been updated.
